# Editorial: Role of non-coding RNAs (emphasis on the emerging role of circular RNAs) in cancer. Application potential for molecular diagnostics and therapeutics of cancer

**DOI:** 10.3389/fgene.2024.1450309

**Published:** 2024-07-25

**Authors:** Srivatsava Naidu, Srikanth Karnati, Harikrishnan Radhakrishnan, Anand Gupta

**Affiliations:** ^1^ Department of Biomedical Engineering, Indian Institute of Technology Ropar, Rupnagar, India; ^2^ Institute of Anatomy and Cell Biology, University of Würzburg, Würzburg, Germany; ^3^ Biosciences Division, SRI International, Menlo Park, CA, United States; ^4^ Department of Dentistry, Government Medical College and Hospital, Chandigarh, India

**Keywords:** non-coding RNAs in cancer, circular RNAs (circRNA), long non-coding RNAs (lncRNA), microRNAs, cancer biology

Amidst the intricate genetic landscape of the human genome, a diverse array of non-coding RNAs (ncRNAs) has emerged as critical regulators of gene expression and cellular function (Nemeth et al., 2024). Among these, circular RNAs (circRNAs), microRNAs (miRNAs), and long non-coding RNAs (lncRNAs) form essential components of regulatory networks that govern cell fate in both normal and pathological contexts (Loganathan and Doss, 2023). There is substantial evidence that dysregulation of these ncRNAs is a hallmark of cancer. Notably, their distinct expression profiles hold immense potential for diagnosis, prognosis, and therapy, opening new avenues for molecular diagnostics and combinatorial treatments (Uppaluri et al., 2023). This editorial summarizes five high-impact review articles that delve into the pivotal advancements of ncRNAs in cancer research, addressing critical aspects of molecular oncology.

Circular RNAs (circRNAs) are a unique class of non-coding RNAs, characterized by their covalently closed loop structures formed by back-splicing of primary transcripts (Zhang et al., 2020). CircRNAs regulate gene expression through various intricate mechanisms, including interactions with nucleic acids and proteins, and significantly influence gene expression at the epigenetic, transcriptional, post-transcriptional, and translational levels (Liu and Chen, 2022). CircRNAs are becoming increasingly important in cancer biology as they regulate key pathways involved in tumorigenesis, exhibit tumor-specific expression patterns, and demonstrate exceptional stability in tumors and bodily fluids, underscoring their significant potential as biomarkers and therapeutic targets (Pisignano et al., 2023).

Zhu et al. critically reviewed the multifaceted roles of circSMARCA5 in cancer, emphasizing its potential as a biomarker. CircSMARCA5, derived from the SMARCA5 gene, exhibits dual roles in tumor suppression and promotion. In gastric cancer, its downregulation correlates with poor prognosis and advanced disease stages, while its overexpression suppresses proliferation and invasion. In hepatocellular carcinoma, low levels of circSMARCA5 are associated with aggressive tumor behavior, whereas its overexpression inhibits tumor growth and metastasis. In glioblastoma, circSMARCA5 interacts with the RNA-binding protein SRSF1 and inhibits cell migration and angiogenesis. Conversely, in cervical, prostate, and bladder cancer, circSMARCA5 can act as a tumor promoter, indicating a context-dependent function. This dualistic nature underscores the complexity of the role of circSMARCA5 in cancer. Additionally, circSMARCA5 is implicated in non-cancerous diseases such as osteoporosis and cardiovascular disease, demonstrating its broad relevance.

Matos et al. conducted a systematic review of the clinicopathological significance of circRNAs in cervical cancer (CC) and identified several circRNAs as potential diagnostic and prognostic markers. CircRNAs like circ_0000730 and circ_0043280 are linked to advanced tumor stages and poorer survival rates, while circ_0018289 exhibits high sensitivity and specificity in distinguishing malignant from benign tissue. These findings underscore the crucial role of circRNAs in CC and their potential to improve diagnostic and prognostic accuracy.

Babayev et al. reviewed advancements in the role of circRNAs in disease mechanisms, diagnosis, and potential therapeutic targets in lung cancer. CircHIPK3 emerged as a key player, promoting cell proliferation and inhibiting apoptosis by sponging miR-124-3p, thereby activating the STAT3 signaling pathway. The review also highlighted circ_0003222’s significant role in chemoresistance by sponging miR-527 and enhancing resistance to cisplatin, a common chemotherapy drug. CircRNAs like circ_0000190, whose plasma levels correlate with tumor progression and treatment response, hold promise as non-invasive biomarkers for early detection and prognosis.

LncRNAs regulate gene expression by serving as molecular scaffolds, guides, decoys, or enhancers by interacting with DNA, RNA, and proteins at various levels (Ulitsky and Bartel, 2013). In cancer, the aberrant expression of lncRNAs is closely linked to tumorigenesis, progression, and metastasis (Qian et al., 2020). Leveraging lncRNAs in clinical applications promises to revolutionize cancer treatment, open new avenues for precision medicine and significantly improve patient outcomes. Lin et al. reviewed the lncRNA BBOX1-AS1 in various cancers, highlighting its significant role in cancer progression through interactions with miRNAs and modulation of key signaling pathways. High BBOX1-AS1 expression is associated with poor overall and disease-free survival, particularly at advanced stages and with increased lymph node metastasis.

MiRNAs are small, non-coding regulatory RNAs that modulate gene expression by binding to complementary sequences in the 3′untranslated regions (3′UTR) of target mRNAs, resulting in mRNA degradation or translational repression. Dysregulation of miRNAs is a hallmark of cancer and plays a critical role in tumor initiation, progression, metastasis, and therapy resistance (Peng and Croce, 2016). Specific miRNA expression profiles can accurately distinguish between different cancer types, predict disease outcomes, and monitor treatment responses, underscoring their diagnostic and prognostic value. MiRNAs are being explored as therapeutic targets, with strategies aimed at inhibiting oncogenic miRNAs (oncomiRs) or restoring the function of tumor-suppressive miRNAs (Naidu et al., 2015).

Wang et al. reviewed the role of miRNAs in the PI3K/AKT pathway and their impact on lung cancer. MiR-425-5p promotes cancer cell proliferation by downregulating PTEN and increasing pathway activity, while miR-379-5p acts as a tumor suppressor by inhibiting the PI3K/AKT pathway. Targeting miRNAs presents significant therapeutic potential, with miR-449a suppressing tumor growth by downregulating NNMT and increasing PTEN expression. Additionally, miRNAs may increase the sensitivity of cancer cells to targeted therapies, paving the way for novel combination treatments.

In conclusion, these articles highlight the critical importance of ncRNAs as biomarkers and therapeutic targets in cancer. They serve as valuable references for the advancement of biomedical research and clinical applications. As research progresses, we anticipate a significant expansion in our understanding and utilization of ncRNAs in clinical settings.
